# Scoop thrombectomy: A declotting technique for the treatment of thrombosed autologous arteriovenous fistula. A single-center retrospective study

**DOI:** 10.1371/journal.pone.0276067

**Published:** 2022-10-13

**Authors:** Lin Ruan, Yanli Yang, Guangwei Ren, Wen Li, Lijun Sun, Lihong Zhang

**Affiliations:** Nephrology Department, Hebei Medical University First Hospital, Shijiazhuang, Hebei, China; University Magna Graecia of Catanzaro, ITALY

## Abstract

**Background:**

Thrombosis is one of the main complications leading to the failure of autologous arteriovenous fistula (AVF) for patients with renal failure. Thrombectomy is one of the major therapies to remove thrombi to salvage the AVF and prolong its patency.

**Materials and methods:**

Fifty-six patients with AVF thrombosis at the anastomosis were recruited for this study and underwent thrombectomy procedures. Their clinical variables were collected. The vasculature was accessed at the site of the aneurysmal dilatation. Under ultrasound guidance, a scoop thrombectomy procedure was performed by anterograde and retrograde scooping to remove the thrombus using forceps. Then, a sheath was placed in the direct vertical direction. Angioplasty was performed with a balloon to treat the underlying primary arteriovenous stenosis. Patients were followed up for 12 months after surgery. The procedural success, primary and secondary patency rates, and incidence of procedure-related complications were analyzed.

**Results:**

There were 2 minor (3.6%) and no major complications. Clinical success was achieved in 55 of the 56 procedures (98.2%). No symptomatic pulmonary embolism or arterial embolization was noted. The primary patency rates at 3, 6, and 12 months were 92.9, 83.8, and 73.3%%, respectively, according to the Kaplan–Meier survival analysis.

**Conclusion:**

Scoop thrombectomy is a safe procedure with high technical success and a low complication rate, and it is an effective method for patients to receive hemodialysis immediately.

## Introduction

In 2015, there were an estimated 2.62 million people with end-stage renal disease (ESRD) worldwide, most of whom received hemodialysis via an arteriovenous fistula (AVF). However, AVF failure is a serious problem, which is caused by venous stenosis formation, thrombosis, and pseudoaneurysm, etc. In particular, thrombosis is a major complication of vascular access. It is mainly caused by obstructive stenosis and needs to be treated as soon as possible. Thrombotic occlusion is usually located at the site of the AV anastomosis. Thrombectomy and pharmacomechanical thrombolysis are the two major approaches to remove thrombi and repair stenotic lesions to salvage the AVF and prolong its patency.

Although it was suggested in the National Kidney Foundation Kidney Disease Outcomes Quality Initiative (NKF KDOQI) that surgical/endovascular thrombectomy and pharmacomechanical thrombolysis were equally effective treatments, both methods have some drawbacks that need to be improved. In fact, some researchers found poor long-term results in patients who received both surgical and endovascular treatments [1, 2]. In a meta-analysis by Tordoir et al., the 1-year primary patency rate was approximately 70–9% after endovascular treatment and approximately 93–51% after surgical treatment [2]. In addition, both surgical and endovascular treatments could result in some complications in 8–10% of patients [3], such as loss of usable arterial and venous segments for establishing future arteriovenous access [4] and hematomas [5]. In addition, intravenous thrombolysis could result in bleeding complications [6], especially for patients within the early postoperative period (<30 days), based on the recommendations of the NKF KDOQI. Therefore, it was suggested in the NKF KDOQI to optimize the current thrombectomy method or develop a more efficient and safer procedure to improve the success and patency rates and minimize the risk of complications. A new treatment method should be performed as soon as possible to avoid central venous catheter placement and to shorten the period of thrombus vessel wall contact [3, 7]. Moreover, this new procedure method should not be viewed as competing but rather complementary to open surgical and endovascular approaches.

In this study, we designed a scoop thrombectomy method using surgical mosquito hemostatic forceps to remove thrombi from a forearm AVF, followed by high-pressure balloon angioplasty. We present a retrospective analysis of our 1-year experience performing 56 AVF declotting procedures.

## Materials and methods

### Participants and study design

This was a single-center retrospective study of the outcome of scoop thrombectomy for the treatment of thrombosed autologous arteriovenous fistula conducted from January 2019 to December 2020. The inclusion criteria of this study included (1) age between 18–80 years and (2) forearm thrombosed AVF combined with stenosis at the anastomosis. The exclusion criteria of the study included (1) puncture site infection, (2) systemic infection, (3) heart failure, (4) brachial AVF, (5) failed to provide written consent, and (6) failure to complete at least 1 follow-up.

In total, 65 patients were recruited in this study. However, 2 patients failed to provide a consent form and were excluded; 3 patients were excluded due to infection at the puncture site; 1 patient was excluded due to heart failure; 1 patient was excluded due to systemic infection; and 2 patients were excluded due to failure to complete any follow-ups. Ultimately, 56 patients who underwent scoop thrombectomy for thrombosed autologous arteriovenous fistula participated in this study.

### Compliance with ethical standards

Study protocols involving human subjects were approved by the First Hospital of Hebei Medical University institutional ethics committee (20190303, Chinese Clinical Trial Registry No. ChiCTR1900021975). All methods were performed in accordance with the relevant guidelines and regulations. The included subjects signed written consent forms agreeing to participate in the study. All data acquired were anonymized.

### Technical notes

**Fig 1:**
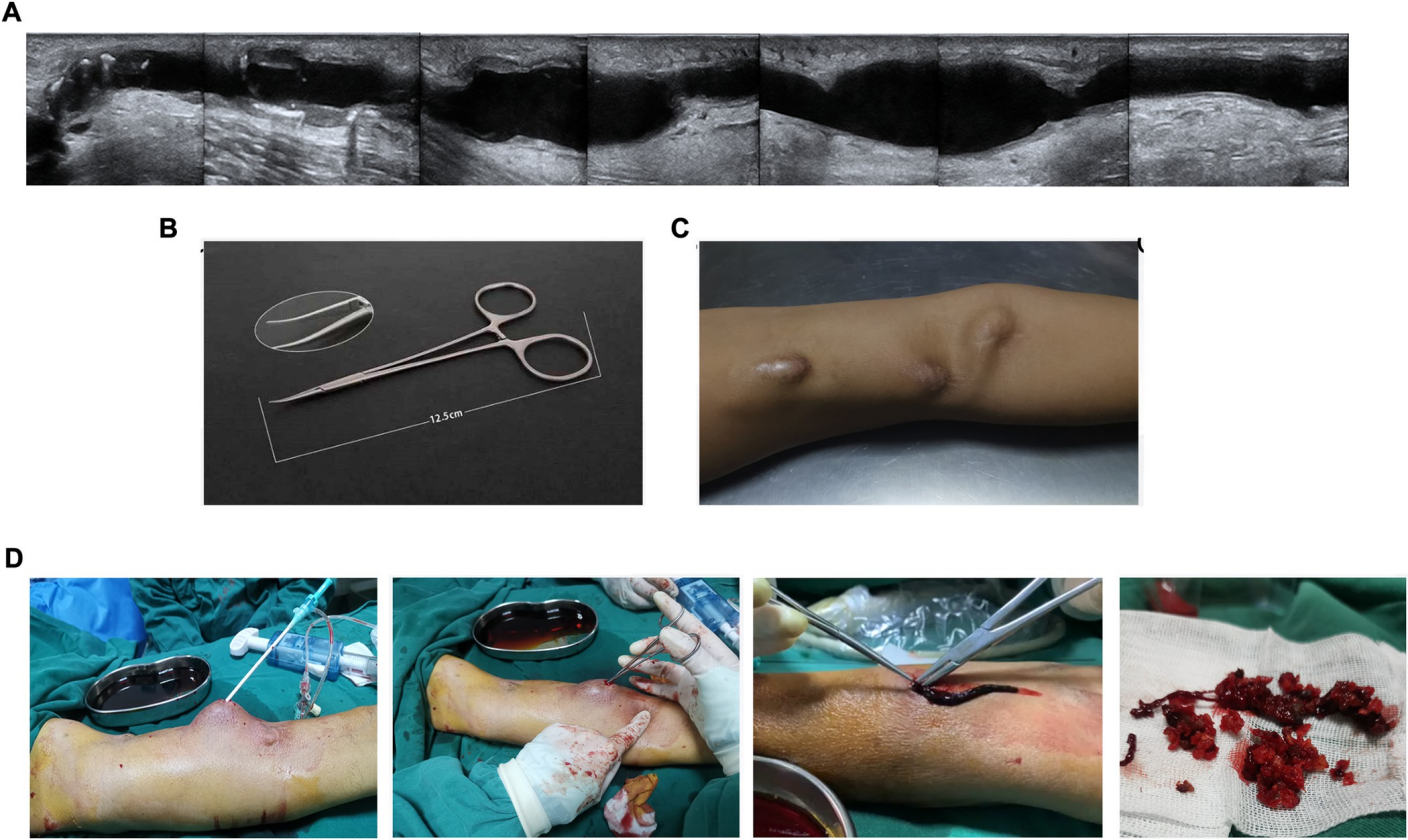
Representative case of scoop thrombectomy. (A) Ultrasound image demonstrating AVF thrombosis at the site of the anastomosis. (B) Mosquito hemostatic forceps were used in the procedure. (C) Aneurysmal dilatation. (D) Surgical procedure. During surgical exploration, the forceps were inserted into the hole of the separated fistula, and the thrombus was removed.

Routine prophylaxis with oral antibiotics was used during the perioperative period. Then, an ultrasound was performed to evaluate the extent of the thrombus and the existence of stenosis. After IV infusion of 2500 IU heparin, the puncture site was chosen near the site of the aneurysmal dilatation. Under ultrasound guidance, vascular access was performed using a needle under regional anesthesia and then dilated using an 8F short sheath. Then, monitoring by ultrasound, the thrombus was scooped out anterogradely and retrogradely using mosquito hemostatic forceps (Fig 1). For thrombi that could not be reached by forceps, an 8F short sheath was placed in the direct vertical direction, and then a regular mechanical thrombectomy was performed to remove the residue of the thrombus. First, a glide wire was inserted into the fistula, then a Fogarty balloon was placed into the AVF along the wire and passed the arterial anastomosis and thrombus. Then, the inflated balloon was pulled back to dislodge the occluding clot.

Once the thrombus had been cleared and blood flow was re-established, an ultrasound was performed to check for stenotic lesions. Under ultrasound, stenotic areas were identified by observation of narrowing at the arteriovenous anastomosis as follows: (1) the diameter of the vein cavity was decreased more than 50% compared to the upstream vein; (2) the ratio of the peak systolic velocity (PSV) of blood flow at this stenosis site to that 2 cm upstream in vein exceeded 3.0 when the stenosis site was within 2 cm of the anastomosis or exceeded 2.0 when the location was more than 2 cm of the anastomosis; (3) in the outflow stenosis: mid PSV < 100 cm/sec, or distal vein > 300 cm/s; and (4) in the inflow stenosis: PSV increased at the site of stenosis with monophasic and diminished waveforms distal.

If a hemodynamically significant stenosis lesion was encountered, an 8F short sheath was placed, and angioplasty of the arteriovenous stenosis was performed with a balloon (Conquest 6×40 mm) to treat the underlying primary stenosis at 30 atm under the guidance of ultrasound. A final ultrasound examination was performed immediately after the angioplasty to determine the success of the procedure.

### Data collection, definitions, and outcomes

Clinical variables and demographic characteristics were extracted from the electronic database of the recruited patients’ medical records, including age, sex, duration of dialysis (months, m), duration of vascular access (months, m), duration of thrombus (hour, h), smoking history, hypertension history, and hyperlipidemia history. Our data contained no personally identifiable information or were pseudonymized through encryption of personal identifiers.

The outcome of the surgery and the AVF function of all the involved patients were followed up for 1 year. The follow-up procedures were conducted in accordance with the Society of Interventional Radiology (SIR) Quality Improvement Guidelines for Percutaneous Image-Guided Management of the Thrombosed or Dysfunctional Dialysis Circuit [3]. The outcomes for follow-up in the present study included (a) primary patency: the interval between postsurgery and the next thrombus or malfunction of the AVF requiring further intervention; (b) clinical success: at least one complete dialysis session through the treated thrombotic AVF after surgery; (c) adverse event: any events related to the procedure; (d) procedure success: a successful completed scoop thrombectomy, followed by a palpable pulse and uninterrupted antegrade flow; and (e) secondary patency: the initial intervention failed to the level of thrombosis and stenosis and was retreated. Once the second treatment was successfully performed, secondary patency defined the durability of that second intervention.

### Statistics

All the data were analyzed in SPSS 26 for statistical computing and graphics. The data of the clinical variables and demographic characteristics are expressed as the mean ± standard deviation (SD) for continuous variables when the data were normally distributed, as a 95% confidence interval when the data had a skewed distribution, or counts (percentages) for discrete variables. Kaplan–Meier survival curve analysis was employed to evaluate some outcomes.

## Results

A total of 56 scoop thrombectomies were performed in the present study (Table 1). The mean procedure time was 68 min. Clinical success was achieved in 55 of the 56 procedures (98.2%), which were successfully used for hemodialysis immediately afterward. In 1 patient (1/56, 1.8%), the procedure failed due to severe calcification at the anastomosis site, so a central catheter was placed for the patient to undergo dialysis, and then another AVF was created on the other side of the forearm. Among the 56 thrombectomies, 54 patients only underwent scooping thrombectomy, and 2 patients underwent scooping thrombectomy combined with mechanical thrombectomy because the thrombus (longer than 3 cm) could not be eliminated by forceps. All 55 patients received angioplasty due to arteriovenous stenosis.

**Table 1:** Demographic data and clinical variables of involved patients’ cohort.

Variable	Stenosis AVF group
Patients, n	56
Age(y)	59.3±12.4
Male(%)	29(51.8)
Duration of dialysis(y)	5.0(4.4, 6.6)
Duration of AVF vascular access (y)	3.5(3.7, 5.6)
Duration of thrombus(h)	24(42.4, 161.9)
Number of procedures	69
Diabetes history(n,%)	18(32.1)
smoke history(n,%)	11(19.6)
hypertension history(n,%)	24(42.9)
hyperlipidemia history(n,%)	10(17.9)

The data of the clinical variables and demographic characteristics are expressed as the mean ± standard deviation (SD) for continuous variables when the data were normally distributed, as a 95% confidence interval when the data had a skewed distribution, or counts (percentages) for discrete variables.

Duration of dialysis: the time interval from the beginning of hemodialysis to the end of present study. Duration of AVF vascular access: the time interval from the created AVFs use for hemodialysis to AVFs failure.

**Fig 2:**
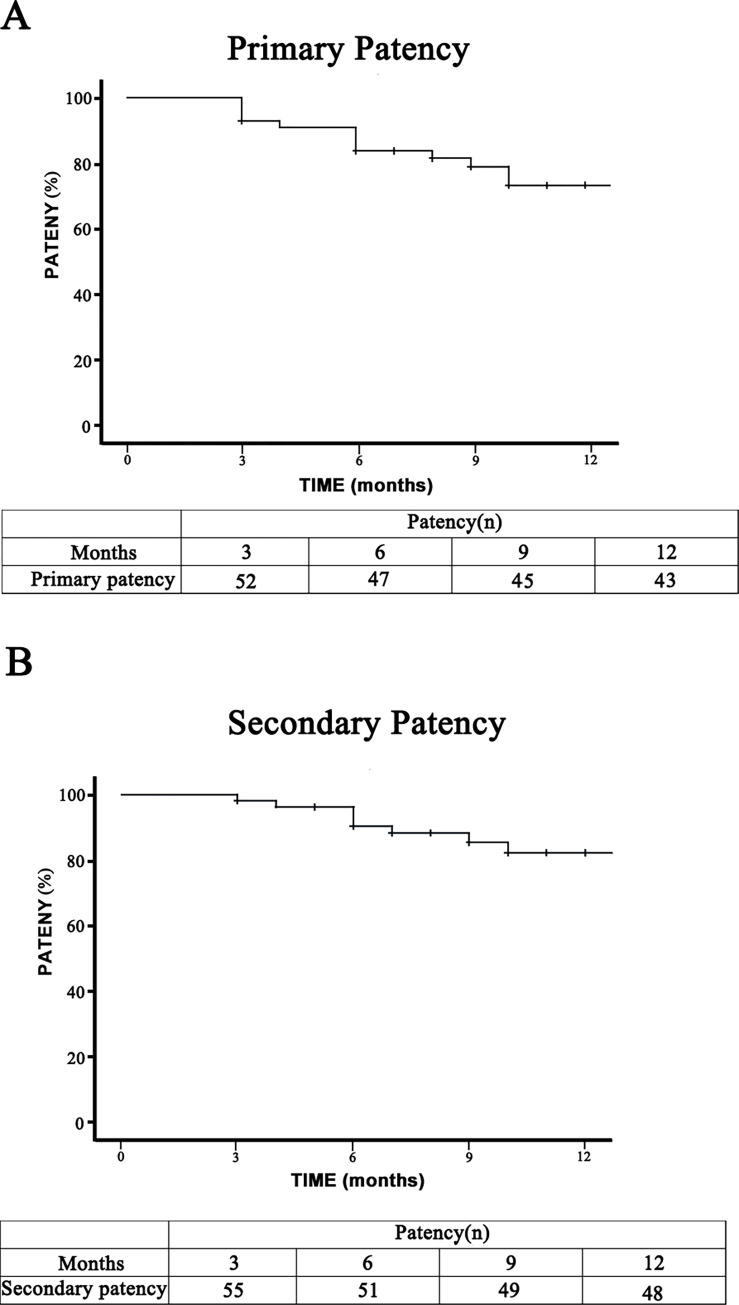
Kaplan–Meier survival analysis curves of scoop thrombectomy procedures in 56 cases. (A). Primary patency. Fifty-six patients underwent scoop thrombectomy procedures in our center, 1 of whom did not achieve primary patency within the treatment period. The initial proportion of patients who achieved primary patency was 98.2%. Thirteen additional patients lost primary patency over the duration of the study. The primary patency probability at 1 year is shown on the curve, and the actuarial primary patency rates at 3, 6, and 12 months are shown below. (B) Secondary patency. Thirteen patients required revision of AVF. Thrombosis occurred in 2 patients who had a revision to restore secondary patency, and stenosis occurred in the 10 patients who had a revision to restore secondary patency. In addition, the angioplasty procedure failed due to severe calcification at the anastomosis site in 1 patient. Secondary patency rates at 3, 6, and 12 months were 98.2%, 90.4%, and 82.3%, respectively. The secondary patency probability at 1 year is shown on the curve.

After surgery, the follow-up period was 1 year. The primary patency rates at 3, 6, and 12 months were 92.9, 83.8, and 73.3%, respectively. The mean time between the intervention for primary access thrombosis and the next access-related event (either surgical revision or clinically verified thrombosis) was 11.2 months (95% confidence interval: 10.4–12.1 months). The crude Kaplan–Meier estimates of patency time for the treatment groups are displayed in Fig 2.

Thirteen additional patients lost primary patency over the duration of the study and required revision of the AVF. Thrombosis occurred in 2 patients who underwent a revision procedure to restore secondary patency, and stenosis occurred in the 10 patients who underwent a revision procedure to restore secondary patency. In addition, the angioplasty procedure failed due to severe calcification at the anastomosis site in 1 patient. The secondary patency rates at 3, 6, and 12 months were 98.2%, 90.4%, and 82.3%, respectively (Fig 2).

Complications occurred in 2 of the 56 patients (3.6%) due to puncture site hematomas. One of them was a small hematoma, a grade 1 complication according to the ASDIN (American Society of Diagnostic and Interventional Nephrology) classification [8]. A large hematoma developed in the other case, resulting in recurrent thrombosis of the fistula and failure of the procedure, and another AVF was subsequently created. These two local complications were related to the guidewire or angioplasty but not to the thrombectomy procedures. No symptomatic pulmonary embolism or arterial embolization was noted.

## Discussion

In the present study, we developed a new thrombectomy strategy to treat AVF thrombolysis, in which we performed thrombus extraction by directly scooping it out. Our data showed that this scoop thrombectomy method is a safe and effective approach to treating thrombosed failing/maturing AVFs.

There are different approaches to declot the thrombosed AVFs, including thrombectomy, thrombolysis, or a ‘‘mix and match” of these procedures. Surgical thrombectomy is a classic method to treat AVF thrombosis. Although open surgery thrombectomy has multiple advantages, such as high success rates and primary patency rates, acknowledged limitations include arterial exposure, vessel injury, and incomplete clot extraction, which could be due to repeated intravascular dislodgement operations [2]. Endovascular thrombectomy carries the risk of vein rupture and failure to complete the treatment due to tight stenosis [2]. Catheter-directed thrombolysis is a minimally invasive therapy method to treat AVF thrombolysis, but significant limitations of lytic therapy exist, including bleeding and intracranial hemorrhage. Previous studies have reported intracranial bleeding rates of approximately 1% [9]. Therefore, to date, the optimal procedure for the treatment of AVF thrombosis remains to be developed. Researchers have focused on the development of surgical instruments for thrombectomy or optimization of the dose/time for thrombolysis.

In the present study, we used standard mosquito hemostatic forceps to extract the thrombus, and then the stenosis was dilated by a balloon. Our results show a technical success rate of 100% in clot removal, which is similar to other studies (range, 76%–100%) [10, 11]. The primary patency rate of our study at 3 months was 92.9%, while the 6-month primary patency was 83.8%.

In the present study, our scoop thrombectomy had multiple advantages, in contrast to traditional methods, including surgical/endovascular thrombectomy and thrombolysis. Some major complications can occur during surgical/endovascular thrombectomy treatment, including hematoma, sepsis, infection, rupture of the vein, pulmonary embolism, or arterial embolization. Researchers in previous studies have demonstrated that in surgical/endovascular thrombectomy of thrombosed AVFs, extended procedure times lead to increased complication rates [12, 13]. However, the mean procedural time in this study was 68 min, which was clearly shorter than the mechanical thrombectomy reported by other studies (126 min; range, 105–160 min). Thus, our scoop thrombectomy minimized the risk of complications of AVF thrombectomy. During mechanical thrombectomy, one of the main risks is vascular injury. Marcelin et al. reported perforation of the vasculature during mechanical/aspiration thrombectomy in acute thrombosis of dialysis arteriovenous fistulae [14], while Kurre et al. suggested that mechanical thrombectomy could result in vascular injury, such as vein rupture [15]. However, in the present study, a guidewire did not need to be inserted during scoop thrombectomy, and the thrombus was scooped out directly, which avoided repeated intravascular procedures during mechanical thrombectomy, such as guidewire insertion, balloon inflation and clot dislodgement, thereby reducing the risk of vascular injury. Using forceps, the thrombus can be removed from the puncture site, which avoids dislodging the thrombus into the central circulation. In contrast, mechanical thrombectomy using a balloon requires insertion of a wire and balloon past the thrombus before removal, which carries the theoretical risk of significant pulmonary embolism or arterial embolization [16].

Researchers have suggested in multiple studies that the learning curve of the surgeon could affect the clinical success of mechanical thrombectomy [17–19]. There is a dose‒response relationship between operator case volume and clinical outcome and procedure time. For example, Baytaroglu et al. suggested that success in the performance of percutaneous thrombectomy for the treatment of acute lower extremity deep vein thrombosis reached satisfactory levels after 20 cases [20]. Although there is a lack of data on the learning curve of AVF mechanical thrombectomy, based on the experience in our clinical center, we suggest that operators perform multiple operations to master the key steps of AVF mechanical thrombectomy, including inserting a guidewire, reaching the thrombus and removing the thrombus using various devices, such as balloons, stents, and aspiration. In contrast, this scooping thrombectomy technique does not require special devices, which shortens the learning curve significantly while maintaining a similar success rate to mechanical thrombectomy or AVF thrombolysis.

In fact, both endovascular thrombolysis and surgical thrombectomy are expensive treatment methods that cost €2097 and €4742, respectively [21], increasing the economic burden for patients and the health care system. In contrast, our scooping thrombectomy does not need expensive/special devices, such as aspiration devices or thrombolytic drugs, which lowers the costs. Furthermore, after scoop thrombectomy, the patients can receive dialysis immediately through the AVF. In contrast, patients who received traditional open surgery had to wait for healing of the lesions before they received dialysis through the AVF. During this window period, those patients must receive central venous catheterization for dialysis, which exposes these patients to extra risks of injury or infection.

In addition, there was only 1 complication during the procedure in the present study, which required more therapy. No symptomatic signs of embolization of thrombus fragments into the system or the pulmonary circulation were noted. Additionally, no bleeding, infection, or vein breakage was found in our study, which indicated the safety of our technology. In contrast, perioperative complications have been reported in the literature to be 0% to 7% with other thrombectomy methods [14, 22, 23].

We must admit that this scooping thrombectomy has some potential drawbacks that need more investigation. First, mechanical thrombectomy might be needed as a complementary treatment to scooping thrombectomy for large thrombi. Based on our preliminary experience, when the thrombus is longer than 3 cm, surgical mosquito hemostatic forceps can barely reach the end of the thrombus, which means that scooping thrombectomy cannot eliminate the thrombus completely. Then, mechanical thrombectomy should be performed following scooping thrombectomy to remove any residual thrombi. Therefore, more research needs to be done to determine the indications for scooping thrombectomy and the treatment standards for scooping devices. Another drawback is the potential risk of vascular wall injury. Theoretically, inappropriate use of the surgical forceps could result in perforation, dissection, or intima injury. Thus, the risk of using a scooping device should be investigated to avoid injury and improve clinical success.

### Limitations

This study was a retrospective analysis from one center. One of the main limitations of this study is the small number of cases and the short follow-up period. We did not involve another thrombectomy method as controls to compare the treatment outcomes. In addition, we only involved cases with forearm-thrombosed AVF at the anastomosis. It remains unclear whether this scoop thrombectomy can be used in thrombosed AVFs under other conditions, such as thrombosis in grafts or brachial AVFs.

Future studies should create a protocol for scoop thrombectomy, such as indications or contraindications for this procedure, by involving more cases, collecting more clinical data from the involved patients, and following them up for a longer period. More clinical studies should be considered to explore the treatment outcome of scoop thrombectomy on thrombosed AVF at sites other than the anastomosis and to compare the treatment outcome between scoop thrombectomy and other thrombectomy methods.

## Conclusion

Scoop thrombectomy was performed to treat AVF thrombosis with high clinical success and patency rates and minimal complications.

## Supporting information

S1 Checklist STROBE statement—checklist of items that should be included in reports of observational studies. (DOCX)
