# Supplementary material for: Scoop thrombectomy: A declotting technique for the treatment of thrombosed autologous arteriovenous fistula. A single-center retrospective study
Source: PLoS One. 2022 Oct 13;17(10):e0276067. doi: 10.1371/journal.pone.0276067 (PMC9562147; doi:10.1371/journal.pone.0276067)
Supplement: S1 Checklist — (DOCX) [file pone.0276067.s001.docx]

STROBE Statement—checklist of items that should be included in reports of observational studies

|  | Item No. | Recommendation | Page  No. | Relevant text from manuscript |
| --- | --- | --- | --- | --- |
| **Title and abstract** | 1 | (*a*) Indicate the study’s design with a commonly used term in the title or the abstract | P1 | A single center's retrospective study/ Follow-up assessments were performed foe 12 months after surgery, |
|  |  | (*b*) Provide in the abstract an informative and balanced summary of what was done and what was found | P1 | 56 Patients with thrombotic AVF at the anastomosis were recruited for this study and had the thrombectomy procedures. Follow-up assessments were performed foe 12 months after surgery./ Postintervention primary patency rates at 3, 6, and 12 months were 92.6, 83.9, and 73.2% according to Kaplan– Meier survival analysis. There were 2 minor (3.6%) and no major complications. Clinical success was achieved in 55 of the 56 procedures (98.2%). No symptomatic pulmonary embolism or arterial embolization was noted. |
| Introduction | | | |  |
| Background/rationale | 2 | Explain the scientific background and rationale for the investigation being reported | P2 | Especially thrombosis is the major drawback of vascular access, caused by obstructive stenoses and needed to be treated as soon as possible. Surgical or endovascular treatments are the two major therapies to remove thrombus and repair of stenotic lesions to salvage AVF and prolong of patency. /more efficient and more safe procedure needs to be developed. |
| Objectives | 3 | State specific objectives, including any prespecified hypotheses | P2 | We herein describe a scoop thrombectomy method using mini surgical thrombectomy combination with high-pressure balloon angioplasty. And in this study, we presented a retrospective analysis of our 1-year experience with 56 AVF declotting procedures. |
| Methods | | | |  |
| Study design | 4 | Present key elements of study design early in the paper | P3 | A single center's retrospective study |
| Setting | 5 | Describe the setting, locations, and relevant dates, including periods of recruitment, exposure, follow-up, and data collection | P3 | Study protocols involving human subjects were approved by the First Hospital of Hebei Medical University institutional ethics committee (20190303, Chinese Clinical Trial Registry No. ChiCTR1900021975). All methods were performed in accordance with the relevant guidelines and regulations. This is a single-center retrospective study that during January 2019-December 2019. All the involved subjects have signed written consent forms to agree to participate the study on their behalf. All data acquired were kept anonymized. |
| Participants | 6 | (*a*) *Cohort study*—Give the eligibility criteria, and the sources and methods of selection of participants. Describe methods of follow-up  *Case-control study*—Give the eligibility criteria, and the sources and methods of case ascertainment and control selection. Give the rationale for the choice of cases and controls  *Cross-sectional study*—Give the eligibility criteria, and the sources and methods of selection of participants | P3 | The inclusion criteria of this study included (1) age between 18-80 and (2) forearm thrombosed AVF at the anastomosis. The exclusion criteria of the study included (1) systemic infection, (2) heart failure, (3) failure to obtain the written consent forms.  Clinical variables and demographic characteristics were collected from the recruited patients, including age, gender, duration of dialysis (months, m), duration of vascular access (months, m), duration of thrombus (hour, h), smoke history, hypertension history, hyperlipidemia history. Our data contained no personally identifiable information or were pseudonymized through encryption of personal identifiers.  Outcome of surgery and the AVF function of all the involved patients were follow up for 1 year by the staff of this study. The follow up procedures were in accordance with the Society of Interventional Radiology (SIR) Quality Improvement Guidelines for Percutaneous Image-Guided Management of the Thrombosed or Dysfunctional Dialysis Circuit [3]. The outcomes for follow up in present study included: (a) postintervention primary patency, the interval between post-surgery and next thrombus, or malfunction of AVF needed further surgical intervention; (b) postintervention clinical patency, at least one complete dialysis session through the treated thrombotic AVF after surgery; (c) adverse event; any events related to the procedure; (d) procedure success, a successful completed Scoop thrombectomy, following with palpable thrill, and uninterrupted antegrade flow. |
|  |  | (*b*) *Cohort study*—For matched studies, give matching criteria and number of exposed and unexposed  *Case-control study*—For matched studies, give matching criteria and the number of controls per case |  |  |
| Variables | 7 | Clearly define all outcomes, exposures, predictors, potential confounders, and effect modifiers. Give diagnostic criteria, if applicable | P3 | The outcomes for follow up in present study included: (a) postintervention primary patency, the interval between post-surgery and next thrombus, or malfunction of AVF needed further surgical intervention; (b) postintervention clinical patency, at least one complete dialysis session through the treated thrombotic AVF after surgery; (c) adverse event; any events related to the procedure; (d) procedure success, a successful completed Scoop thrombectomy, following with palpable thrill, and uninterrupted antegrade flow. |
| Data sources/ measurement | 8* | For each variable of interest, give sources of data and details of methods of assessment (measurement). Describe comparability of assessment methods if there is more than one group | P3 | Clinical variables and demographic characteristics were collected from the recruited patients, including age, gender, duration of dialysis (months, m), duration of vascular access (months, m), duration of thrombus (hour, h), smoke history, hypertension history, hyperlipidemia history. Our data contained no personally identifiable information or were pseudonymized through encryption of personal identifiers.  Outcome of surgery and the AVF function of all the involved patients were follow up for 1 year by the staff of this study. |
| Bias | 9 | Describe any efforts to address potential sources of bias | NA |  |
| Study size | 10 | Explain how the study size was arrived at | P3 | This is a single-center retrospective study that during January 2019-December 2019. All the involved subjects have signed written consent forms to agree to participate the study on their behalf. All data acquired were kept anonymized. |

Continued on next page

| Quantitative variables | 11 | Explain how quantitative variables were handled in the analyses. If applicable, describe which groupings were chosen and why | P4 | Data of clinical variables and demographic characteristics were expressed as the mean ± standard deviation (SD) for continuous variables, or counts (percentages) for discrete variables. |
| --- | --- | --- | --- | --- |
| Statistical methods | 12 | (*a*) Describe all statistical methods, including those used to control for confounding | P4 | All the data were analyzed in SPSS 26 for statistical computing and graphics. |
|  |  | (*b*) Describe any methods used to examine subgroups and interactions | P4 | Kaplan–Meier life-table analysis was employed to evaluate outcomes. |
|  |  | (*c*) Explain how missing data were addressed | NA |  |
|  |  | (*d*) *Cohort study*—If applicable, explain how loss to follow-up was addressed  *Case-control study*—If applicable, explain how matching of cases and controls was addressed  *Cross-sectional study*—If applicable, describe analytical methods taking account of sampling strategy | NA |  |
|  |  | (*e*) Describe any sensitivity analyses |  |  |
| Results | | | | |
| Participants | 13* | (a) Report numbers of individuals at each stage of study—eg numbers potentially eligible, examined for eligibility, confirmed eligible, included in the study, completing follow-up, and analysed | P4 | A total of 56 cases of scoop thrombectomies were performed in present study period (Table 1). The mean procedure time was 68 min. Clinical success was achieved in 55 of the 56 procedures (98.2%). |
|  |  | (b) Give reasons for non-participation at each stage | P4 | In 1 case (1/56, 1.8%), procedure failed due to severe calcification at the anastomosis site, and a central catheter was placed for the patient to undergo dialysis. In total, 55 accesses (98.21%) achieved sustained dialysis use after thrombectomy. |
|  |  | (c) Consider use of a flow diagram | NA |  |
| Descriptive data | 14* | (a) Give characteristics of study participants (eg demographic, clinical, social) and information on exposures and potential confounders | Table1 | Table1 |
|  |  | (b) Indicate number of participants with missing data for each variable of interest | P4 | A total of 56 cases of scoop thrombectomies were performed in present study period (Table 1). The mean procedure time was 68 min. Clinical success was achieved in 55 of the 56 procedures (98.2%). In 1 case (1/56, 1.8%), procedure failed due to severe calcification at the anastomosis site, and a central catheter was placed for the patient to undergo dialysis. In total, 55 accesses (98.21%) achieved sustained dialysis use after thrombectomy. |
|  |  | (c) *Cohort study*—Summarise follow-up time (eg, average and total amount) | P4 | After surgery, the follow-up period was 1 year. The primary patency rates at 3, 6, and 12 months were 92.9, 83.8, and 73.3%, respectively. The mean time between the intervention for primary access thrombosis and the next access-related event (either surgical revision or clinically verified thrombosis) was 11.2 months (95% confidence interval: 10.4–12.1 months). The crude Kaplan–Meier estimates of patency time for the treatment groups are displayed in Figure 2. |
| Outcome data | 15* | *Cohort study*—Report numbers of outcome events or summary measures over time | P4 | *After surgery, the follow-up period was 1 year. The primary patency rates at 3, 6, and 12 months were 92.9, 83.8, and 73.3%, respectively. The mean time between the intervention for primary access thrombosis and the next access-related event (either surgical revision or clinically verified thrombosis) was 11.2 months (95% confidence interval: 10.4–12.1 months). The crude Kaplan–Meier estimates of patency time for the treatment groups are displayed in Figure 2.*  *Thirteen additional patients lost primary patency over the duration of the study and required revision of the AVF. Thrombosis occurred in 2 patients who underwent a revision procedure to restore secondary patency, and stenosis occurred in the 10 patients who underwent a revision procedure to restore secondary patency. In addition, the angioplasty procedure failed due to severe calcification at the anastomosis site in 1 patient. The secondary patency rates at 3, 6, and 12 months were 98.2%, 90.4%, and 82.3%, respectively (Figure 2).* |
|  |  | *Case-control study—*Report numbers in each exposure category, or summary measures of exposure | P4 |  |
|  |  | *Cross-sectional study—*Report numbers of outcome events or summary measures | P4 |  |
| Main results | 16 | (*a*) Give unadjusted estimates and, if applicable, confounder-adjusted estimates and their precision (eg, 95% confidence interval). Make clear which confounders were adjusted for and why they were included |  |  |
|  |  | (*b*) Report category boundaries when continuous variables were categorized |  |  |
|  |  | (*c*) If relevant, consider translating estimates of relative risk into absolute risk for a meaningful time period |  |  |

Continued on next page

| Other analyses | 17 | Report other analyses done—eg analyses of subgroups and interactions, and sensitivity analyses | P4 | Complications occurred in 2 of the 56 patients (3.6%) due to puncture site hematomas. One of them was a small hematoma, a grade 1 complication according to the ASDIN (American Society of Diagnostic and Interventional Nephrology) classification [8]. A large hematoma developed in the other case, resulting in recurrent thrombosis of the fistula and failure of the procedure, and another AVF was subsequently created. These two local complications were related to the guidewire or angioplasty but not to the thrombectomy procedures. No symptomatic pulmonary embolism or arterial embolization was noted. |
| --- | --- | --- | --- | --- |
| Discussion | | | | |
| Key results | 18 | Summarise key results with reference to study objectives | P4 | In present study, we developed a new thrombectomy strategy to treat AVF thrombolysis, in which we performed thrombus extraction by scooping out directly. And our data showed that this scoop thrombectomy method is a safe and effective method of treatment for thrombosed failing/maturing AVFs. |
| Limitations | 19 | Discuss limitations of the study, taking into account sources of potential bias or imprecision. Discuss both direction and magnitude of any potential bias | P6 | Limitations  This study was a retrospective analysis from one center. One of the main limitations of this study is the small number of cases and the short follow-up period. We did not involve another thrombectomy method as controls to compare the treatment outcomes. In addition, we only involved cases with forearm-thrombosed AVF at the anastomosis. It remains unclear whether this scoop thrombectomy can be used in thrombosed AVFs under other conditions, such as thrombosis in grafts or brachial AVFs. |
| Interpretation | 20 | Give a cautious overall interpretation of results considering objectives, limitations, multiplicity of analyses, results from similar studies, and other relevant evidence | P5 |  |
| Generalisability | 21 | Discuss the generalisability (external validity) of the study results | P6 | Future studies should create a protocol for scoop thrombectomy, such as indications or contraindications for this procedure, by involving more cases, collecting more clinical data from the involved patients, and following them up for a longer period. More clinical studies should be considered to explore the treatment outcome of scoop thrombectomy on thrombosed AVF at sites other than the anastomosis and to compare the treatment outcome between scoop thrombectomy and other thrombectomy methods. |
| Other information | |  | | |
| Funding | 22 | Give the source of funding and the role of the funders for the present study and, if applicable, for the original study on which the present article is based | P1 | This Project was supported by the Key Research Program of Hebei Province, China (Grant No.21377747D) |

*Give information separately for cases and controls in case-control studies and, if applicable, for exposed and unexposed groups in cohort and cross-sectional studies.

**Note:** An Explanation and Elaboration article discusses each checklist item and gives methodological background and published examples of transparent reporting. The STROBE checklist is best used in conjunction with this article (freely available on the Web sites of PLoS Medicine at http://www.plosmedicine.org/, Annals of Internal Medicine at http://www.annals.org/, and Epidemiology at http://www.epidem.com/). Information on the STROBE Initiative is available at www.strobe-statement.org.
